# Characterization of *Shigella flexneri* in northern Vietnam in 2012–2016

**DOI:** 10.1099/acmi.0.000493.v4

**Published:** 2023-06-08

**Authors:** Dong Tu Nguyen, Masatomo Morita, Tuan Cuong Ngo, Thanh Huong Le, Dang Hai Le, Hoai Thu Nguyen, Yukihiro Akeda, Makoto Ohnishi, Hidemasa Izumiya

**Affiliations:** ^1^​ Department of Bacteriology, National Institute of Hygiene and Epidemiology, Hanoi, Vietnam; ^2^​ Department of Bacteriology I, National Institute of Infectious Diseases, Tokyo, Japan; ^3^​ Bach Mai Hospital, Hanoi, Vietnam

**Keywords:** shigellosis, *Shigella flexneri*, phylogroup (PG) 3, whole genome sequencing, Northern Vietnam

## Abstract

**Introduction.:**

Shigellosis remains a considerable public health concern in developing countries. *Shigella flexneri* and *Shigella sonnei* are prevalent worldwide and *S. sonnei* has been replacing *S. flexneri *.

**Gap Statement.:**

*
S. flexneri* still causes outbreaks of shigellosis in northern Vietnam but limited information is available on its genetic characteristics.

**Aim.:**

This study aimed to characterize the genetic characteristics of *S. flexneri* strains from northern Vietnam.

**Methodology.:**

This study used 17 isolates from eight incidents, collected in northern Vietnam between 2012 and 2016. The samples were subjected to whole genome sequencing, molecular serotyping, cluster analysis and identification of antimicrobial resistance genes. Additionally, phylogenetic analysis was performed including isolates from previous studies.

**Results.:**

Clusters were identified according to spatiotemporal backgrounds. The results suggested that two incidents in Yen Bai province in 2015 and 2016 were derived from a very recent common ancestor. All isolates belonged to phylogroup (PG) 3, which was divided into two sub-lineages. Thirteen of 17 isolates, including those from the Yen Bai incidents, belonged to sub-lineage Sub-1 and were serotyped as 1a. The remaining four isolates belonged to sub-lineage Sub-2 and were the globally predominant serotype 2a. The Sub-1 *S. flexneri* isolates possessed the *gtrI* gene, which encodes the glycosyl transferase that determines serotype 1a, with bacteriophage elements in the vicinity.

**Conclusion.:**

This study revealed two PG3 sub-lineages of *S. flexneri* in northern Vietnam, of which Sub-1 might be specific to the region.

## Data Summary

The data that support the findings of this study are indicated in this manuscript.

## Introduction

*Shigella* is a Gram-negative, facultatively anaerobic, non-spore-forming, and non-motile bacterial genus belonging to the family *Enterobacteriaceae *; it is the aetiological agent of bacillary dysentery or shigellosis. *Shigella* is a common cause of diarrhoea in developing countries, leading to an estimated 269 million episodes and 212 000 deaths annually across all ages worldwide [1]. *Shigella* comprises four species: *Shigella dysenteriae *, *S. flexneri *, *S. boydii* and *S. sonnei *. Approximately 93 % of shigellosis cases are attributed to *S. flexneri* and *S. sonnei* [2]. The ratio of the two species differs among countries; the proportion of *S. flexneri* is higher in developing countries [3]. *S. flexneri* can be subdivided into serotypes based on the O-antigen component of LPS. There are six serogroups, 1–6, and serogroups 1–5 are further subdivided into serotypes such as 2a [4]. Phylogenetically, *S. flexneri* has two distinct lineages, one containing only *S. flexneri* serotype 6 and the other containing all other serotypes. The latter consist of seven phylogroups (PGs) that were determined by single nucleotide polymorphisms [5]. PGs 1–3 are dominant, accounting for 81 %. Although there are dominant serotypes in some PGs (e.g. serotype 1b, 3a and 2a for PG1, PG2 and PG3, respectively), each PG includes multiple serotypes as PGs are not dependent on serotypes. *S. flexneri* serotype 2a of PG3 is predominant globally [5]. *S. sonnei* has been increasing in prevalence and is replacing *S. flexneri *, even in developing countries, including in Southeast Asia [3, 6]. In Vietnam, this species replacement is significant in the southern region [7, 8]. However, shigellosis outbreaks due to *S. flexneri* continue to occur, and information on the genomic characterization remains limited. This study aimed to analyse *S. flexneri* isolates from incidents (sporadic cases and outbreaks) in northern Vietnam using whole genome sequencing (WGS) to uncover the characteristics of *S. flexneri* in this region.

## Methods

### Bacterial isolates

Seventeen *S. flexneri* isolates collected in northern Vietnam between 2012 and 2016 were used in this study. All were isolated in investigations notified by the local health centres, whose information had been anonymized. Sporadic cases and outbreak-related cases were probably included but detailed information was not available. We therefore use the term ‘incident’. A summary of the isolates is presented in Table 1. The reference sequence data used in this study were ERR048305 for PG1, ERR048281 for PG2, AE014073 for PG3, ERR127046 for PG4, ERR832464 for PG5, ERR0848288 for PG6 and ERR048317 for PG7 [5, 9, 10].

**Table 1.** List of isolates used in this study

Isolate	Year	Province	Serotype	Incident	Accession
P12012	2012	Lao Cai	2a	A	BSBP01000000
P12048	2012	Son La	1a	B	BSBQ01000000
P12049	2012	Son La	1a	B	BSBR01000000
P14013	2014	Dien Bien	1a	C	BSBS01000000
P14014	2014	Dien Bien	1a	C	BSBT01000000
P14015	2014	Son La	2a	D	BSBU01000000
P15004	2015	Yen Bai	1a	E	BSBV01000000
P15018	2015	Yen Bai	1a	E	BSBW01000000
P15020	2015	Yen Bai	1a	E	BSBX01000000
P15021	2015	Yen Bai	1a	E	BSBY01000000
P15022	2015	Yen Bai	1a	E	BSBZ01000000
P15023	2015	Yen Bai	1a	E	BSCA01000000
P16001	2016	Yen Bai	1a	F	BSCB01000000
P16002	2016	Yen Bai	1a	F	BSCC01000000
P16018	2016	Cao Bang	2a	G	BSCD01000000
P16020	2016	Cao Bang	2a	G	BSCE01000000
P16026	2016	Son La	1a	H	BSCF01000000
Reference*	**Year**	**Country**	**Serotype**	**PG**†	**Accession**
IB0719	1982	Korea	1b	1	ERR048305
IB1685	na	Vietnam	3a	2	ERR048281
2457T	na	na	2a	3	AE014073
531/61	1961	UK	X	4	ERR127046
CIP 67-61	1966	Iraq	5a	5	ERR832464
IB4235	2000	India	Y	6	ERR048288
IB0017	1991	Korea	Y	7	ERR048317
H04/HUE04	2008	Vietnam	1a	3	ERS031840
H10/HUE10	2008	Vietnam	1a	3	ERS031846
IB0034	2002	China	1a	3	ERS033371
0228	2002	China	1a	3	CP012735

*Reference strain used for phylogenetic analysis.

†Phylogroup.

### Molecular typing and data analyses

The isolates were subjected to WGS. WGS data were obtained using the MiSeq platform (Illumina). Library preparation, genome assembly and single-nucleotide variant (SNV) extraction were performed as described previously [11]. Genomic libraries were prepared by the Nextera XT DNA Library Preparation Kit (Illumina) and sequenced on the MiSeq sequencer (Illumina). Genomic assembly was performed using SPAdes version 3.13.0 (RRID:SCR_000131) with the –careful option and a read coverage cutoff value of 10 [12]. The assembled contigs were incorporated into the BioNumerics software version 8.1 (Applied Maths) for core genome multilocus sequence typing (cgMLST) analysis and for identifying the antimicrobial resistance genes. cgMLST of BioNumerics includes 2513 loci extracted with the Core (Enterobase) setting from 14 837 whole genome MLST (wgMLST) loci (https://www.bionumerics.com/sites/default/files/extra/Release-Note-Eschericha-coli-Shigella-schema.pdf). A minimum similarity of 77.5 % was used for allele calls. Identification of antimicrobial resistance genes was performed using the *Escherichia coli* functional genotyping plug-in of the BioNumerics software with default settings (minimum identity of 95.0 % and minimum coverage of 95.0 % for blast; https://www.bionumerics.com/applications/functional-genotyping). A bio-neighbour joining tree was created by the BioNumerics software with the default settings (using the character data of cgMLST alleles as categorical, Merge taxa when distance is zero and No resampling). Molecular serotyping was performed using ShigaTyper version 2.0.2 and *in silico* PCR screening [13, 14]. SNVs were identified using Snippy version 4.3.6 (https://github.com/tseemann/snippy) using the assembled sequences. *S. flexneri* strain 2457^T^ (accession no. AE014073), which is of serotype 2a and belongs to PG3, was used as a reference. SNVs in recombinogenic, repeat and prophage regions identified by Gubbins version 2.3.1 [15], NUCmer version 4.0.0rc1 [16] and phaster [17] were excluded. A maximum-likelihood phylogenetic tree was generated using RaxML version 8.2.12 [18] with -m GTRGAMMA option and 1000 bootstrap iterations. Sequence comparisons were performed using blastn and visualized using Easyfig [19]. Visualization of overall comparison of whole genome sequences were done using brig [20].

### Sequence data

Nucleotide sequence data have been submitted to the DNA Data Bank of Japan (DDBJ) Sequence Read Archive under accession numbers DRR298131–DRR298147. The data have been deposited with links to DDBJ BioProject accession number PRJDB11754 in DDBJ. The assembled sequence data used in this study have been also deposited to DDBJ. Accession numbers are shown in Table 1.

## Results and discussion

### Clusters found in WGS analysis

**Fig. 1.**
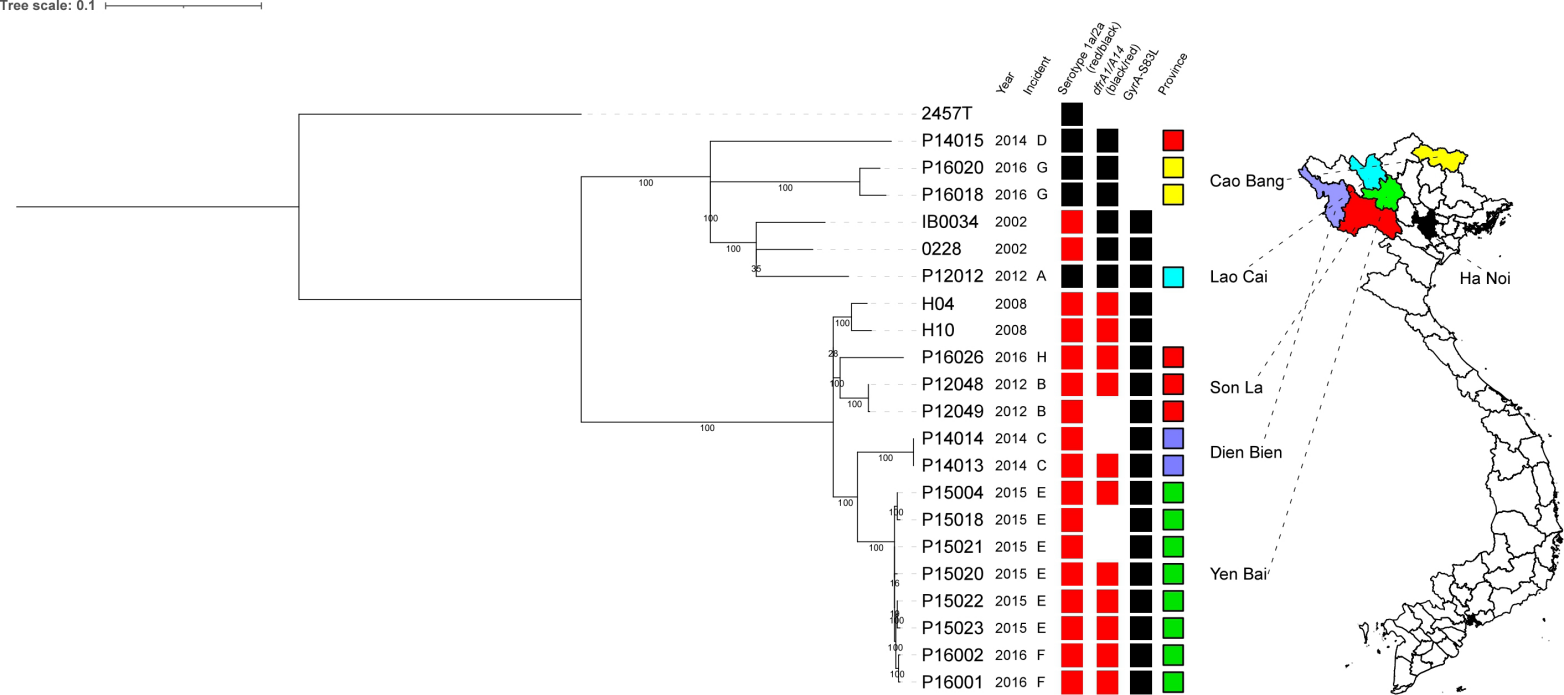
Midpoint-rooted maximum-likelihood phylogeny based on SNVs. The model of GTRGAMMA was used to produce the tree. Boxes indicate serotype (1a/2a), *dfr* genes (*dfrA1*/*dfrA14*), GyrA–S83L mutation and provinces; serotype 1a and *dfrA14* are coloured in red. The location of the province is shown on the right.

WGS analysis created clusters of isolates according to the spatiotemporal backgrounds (Fig. 1). The number of SNVs within each incident was within six, except for incident G of Cao Bang in 2016. One cluster comprised incident B of Son La in 2012. Another cluster comprised incident C of Dien Bien in 2014. The third comprised incidents E and F of Yen Bai in 2015 and 2016, respectively. Although the isolates of Yen Bai in 2016 formed a small branch in SNV analysis (Fig. 1), the number of SNVs was within six. This suggests that the two incidents might have been caused by a single source of contamination.

Two isolates from incident G of Cao Bang in 2016 were assigned next to each other in WGS analyses, although the number of variations was higher than in other clusters (31 SNVs).

### Phylogroup, molecular serotyping and antimicrobial resistance genes

**Fig. 2.**
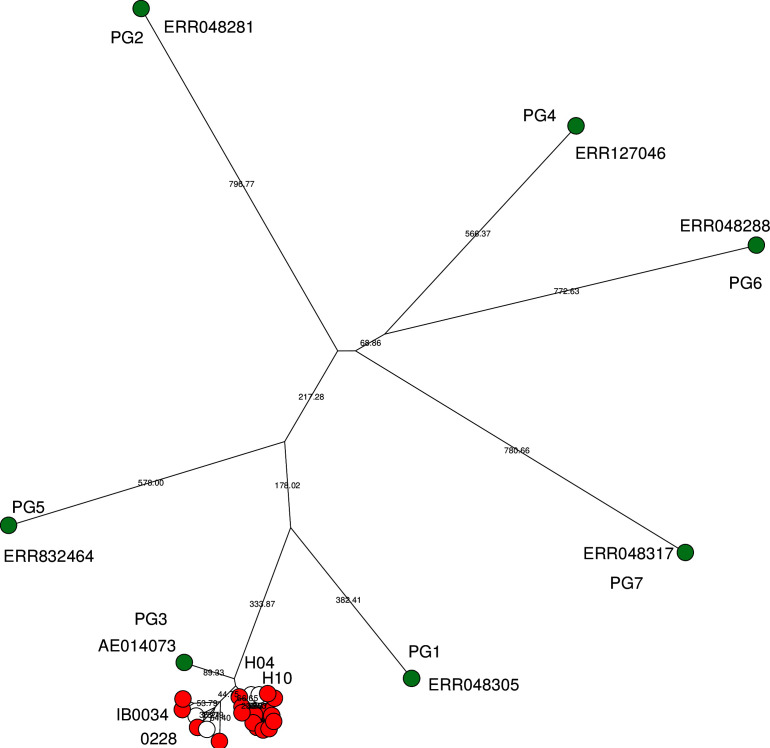
Bio-neighbour-joining tree based on cgMLST. Reference strains of phylogroups are shown as green nodes with accession numbers. Red nodes indicate the isolates in this study. White nodes indicate the PG3 serotype 1a isolates of a previous study [5]. Branch length was calculated based on allele differences.

The isolates in this study were compared to representative strains of the phylogroups [6]. This suggested that all isolates in this study belonged to PG3 (Fig. 2). PG3 is the dominant phylogroup. The isolates could be divided into two sub-lineages in SNV analyses, named Sub-1 and Sub-2 (Fig. 1). Based on molecular serotyping, 13 and four of 17 isolates were serotypes 1a and 2a, respectively. The distribution of serotypes of the 17 isolates in this study completely matched the two sub-lineages, and serotype 1a and 2a isolates belonged to Sub-1 and Sub-2, respectively. According to a previous study, PG3 includes multiple serotypes but serotype 2a is predominant followed by serotype 2b. Serotype 1a is the minor serotype and distributed in PG1 and PG3. PG3 with serotype 1a is quite minor, accounting for only 3 % of PG3 [5]. Data from three PG3 serotype 1a isolates from a previous study [5] were incorporated into the analyses. The three isolates, two from Vietnam (H04 and H10) and one from China (IB0034), were assigned to Sub-1 and Sub-2, respectively. Some antimicrobial resistance genes were also distributed specifically in the sub-lineages (Table 2). For example, *dfrA1* was identified only in Sub-2 while *dfrA14* was identified in Sub-1. All Sub-1 had the GyrA–S83L mutation and *bla*_TEM-1B_. In Sub-2, only one isolate in this study, P12012, had the GyrA–S83L mutation. No other types of *gyrA* mutation were found in this study. A recent study identified two sub-lineages in PG3, Lin-3.1 and Lin-3.2 [8]. The resistance gene for trimethoprim is one of the key features for each Lin; Lin-3.2 was associated with *dfrA1* while Lin-3.1 independently acquired a plasmid-borne *dfrA14*. Another feature discriminating between Lin-3.1 and Lin-3.2 is quinolone resistance. Quinolone resistance emerged in the mid-1990s both in Lin-3.1 and in Lin-3.2, and became prevalent in the mid-2010s in Lin-3.1 but not in Lin-3.2 [8]. These features corresponded to those of the sub-lineages found in this study. Distributions of *dfrA14* and quinolone resistance point mutations were significantly observed in Sub-1. Additionally, *dfrA1* was only found in Sub-2. Thus, it was likely that Sub-1 and Sub-2 corresponded to Lin-3.1 and Lin-3.2.

**Table 2.** Resistance genes/point mutations found in isolates used in this study*

Isolate	Incident	Serotype	Sub-lineage	Resistance gene										Point mutation	
				*aadA1*	*aph(3′)-Ib*	*aph(6)-Id*	*blaOXA-1*	*blaTEM-1B*	*sul2*	*dfrA1*	*dfrA14*	*catA1*	*tet(B*)	GyrA S83L	ParC S80I
P12012	A	2a	2	−	−	−	+	−	+	+	−	+	+	+	+
P12048	B	1a	1	−	−	−	+	+	+	−	+	+	+	+	−
P12049	B	1a	1	−	−	−	+	+	+	−	−	+	+	+	−
P14013	C	1a	1	−	−	−	+	+	+	−	+	+	+	+	−
P14014	C	1a	1	−	−	−	+	+	+	−	−	+	+	+	−
P14015	D	2a	2	−	−	−	+	−	+	+	−	+	+	−	−
P15004	E	1a	1	−	+	+	−	−	+	−	+	−	−	+	−
P15018	E	1a	1	+	+	+	+	+	+	−	−	+	+	+	−
P15020	E	1a	1	−	+	+	−	+	+	−	+	−	−	+	−
P15021	E	1a	1	+	+	+	+	+	+	−	−	+	+	+	−
P15022	E	1a	1	+	+	+	+	+	+	−	+	+	+	+	−
P15023	E	1a	1	+	+	+	+	+	+	−	+	+	+	+	−
P16001	F	1a	1	+	+	+	+	+	+	−	+	+	+	+	−
P16002	F	1a	1	+	+	+	+	+	+	−	+	+	+	+	−
P16018	G	2a	2	−	−	−	+	−	+	+	−	+	+	−	−
P16020	G	2a	2	−	−	−	+	−	+	+	−	+	+	−	−
P16026	H	1a	1	−	−	−	+	+	+	−	+	+	+	+	−

*Plus and minus signs indicate positive and negative for the gene/mutation, respectively.

### Genetic background of serotype 1a isolates

**Fig. 3.**
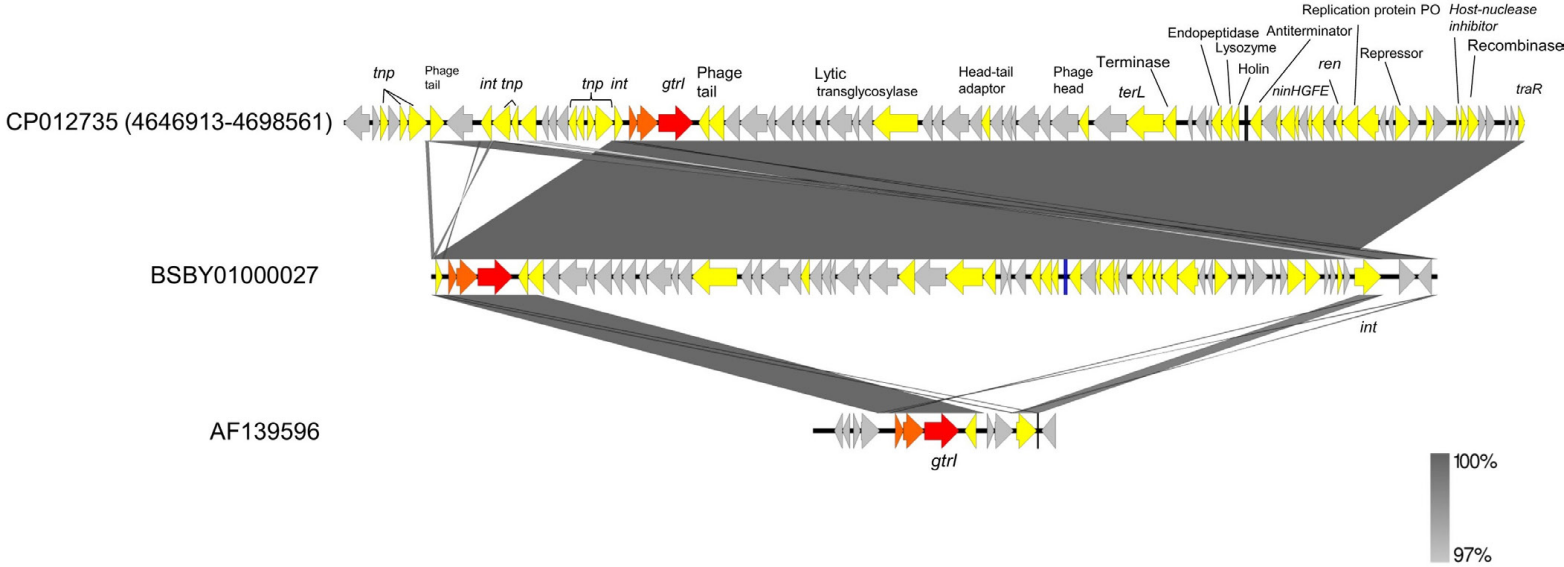
Comparison among genomic regions encompassing *gtrI.* Compared sequences were obtained from: *S. flexneri* 0228 (accession number: CP012735, upper part of the figure), *S. flexneri* P15021 (accession number: BSBY01000027, middle) and *S. flexneri* Y53 (accession number: AF139596, lower). Red arrows, *gtrI*; orange arrows, other *gtr*; blue bars, tRNA; yellow arrows, mobile genetic elements, such as transposons, integrase and bacteriophage-related genes, whose gene/product is indicated in the upper part. Bands connecting the sequences represent levels of nucleotide identity, as indicated in the key in the bottom right.

**Fig. 4.**
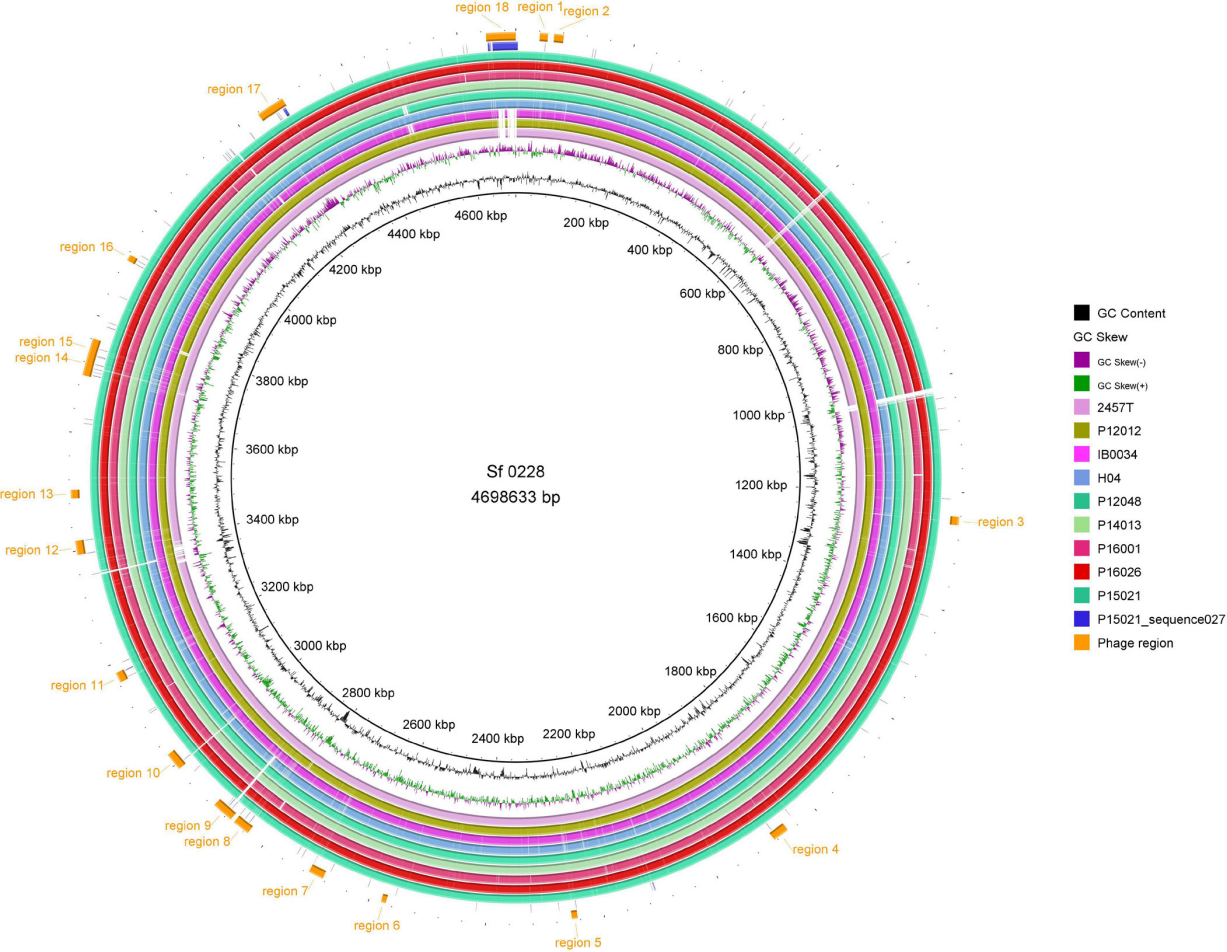
Whole genome sequence alignment of *S. flexneri *. Strain 0228 was used as a reference. The rings shown are, from inside to outside, GC content, GC skew, blast comparisons of *S. flexneri* 2457^T^, P12012, IB0034, H04, P12048, P14013, P16001, P16026, P15021 and P15021 contig BSBY01000027 against the *S. flexneri* 0228 genome, and phage regions identified by phaster [17] (orange).

The O-antigen modification gene responsible for serotype 1a is *gtrI*, which encodes a glycosyl transferase [21]. A 43 kb contig sequence (BSBY01000027) encompassing *gtrI* was identified in P15021. Contig BSBY01000027 was subjected to a blastn search. The top hit sequence was CP012735 of strain 0228 originating from China (coverage 100 % and identity 99.98 %), but not the original sequence of a *gtrI*-containing genetic element, AF139596 (Fig. 3). The matched region of CP012735 contained sequences of *gtrI*, integrase and putative bacteriophage components. Overall comparison between the whole genome sequences indicated that the *gtrI*-containing contig of P15021 corresponded to phage region 18 of strain 0228, which was lacking in the PG3 serotype 2a reference strain 2457^T^ (Fig. 4).

Strain 0228 was assigned to Sub-2 (Fig. 2). Thus, all serotype 1a isolates from Vietnam were assigned to Sub-1, whereas those from China (IB0034 and 0228) were assigned to Sub-2. A serotype 2a isolate, P12012, was assigned in the same branch as strains IB0034 and 0228. This branch is intriguing because multiple serotypes 1a and 2a were included and the phage region 18 responsible for serotype conversion was not conserved in strain IB0034 (Fig. 4). This suggests multiple ways to acquire serotype conversion.

Seroconversion is a major strategy for survival by evading the immune response of the host. Notably, *S. flexneri* 1a isolates of Vietnam were specifically assigned to Sub-1, including those of another study (H04 and H10) [5]. All the representative isolates of serotype 1a isolates in this study and the previous study had the common phage region 18 of strain 0228 identified by phaster [17] (Fig. 4). PG3 predominantly consists of serotype 2a. Serotype 1a isolates in this study were considered to be a quite minor part of PG3. It is difficult to conclude where and when acquisition of the genetic element for serotype 1a occurred because the number of isolates analysed was limited. Nevertheless, this study indicates that Vietnamese isolates of serotype 1a created a specific sub-lineage where the putative seroconversion phage was conserved, suggesting its circulation in northern Vietnam. Further surveillance would be needed to uncover the distribution of *S. flexneri* in Vietnam.

In conclusion, *S. flexneri* infection remains a public concern in northern Vietnam. Clusters of *S. flexneri* were observed based on WGS analysis. All isolates used in this study belonged to PG3 and consisted of two sub-lineages where serotypes 1a and 2a were distributed specifically.
